# A case of functional vitamin B12 deficiency after recreational nitrous oxide use

**DOI:** 10.11613/BM.2024.010801

**Published:** 2023-12-15

**Authors:** Damien Leleu, Damien Denimal

**Affiliations:** 1 Department of Clinical Biochemistry, Dijon University Hospital, Dijon, France; 2 National institute for health and medical research, University of Burgundy, Dijon, France

**Keywords:** vitamin B12, nitrous oxide, homocysteine, methylmalonic acid

## Abstract

The recreational use of nitrous oxide as laughing gas becomes a real public health issue among adolescents and young adults. Chronic use is deleterious and can lead to severe neurological disorders. Nitrous oxide inactivates vitamin B12, and the functional defect of vitamin B12 plays a major role in the pathogenesis of nitrous oxide-related neurological disorders. Here we report the case of a 22-year-old woman who came to the hospital after an unexplained loss of consciousness. She exhibited typical features of vitamin B12 or folate deficiency such as macrocytic anemia and hypersegmented neutrophils. However, serum concentrations of folate and vitamin B12 were normal. In contrast, circulating concentrations of total homocysteine and methylmalonic acid were significantly increased. These results clearly indicated a defect in vitamin B12 functions. The reason for this defect was clarified when she revealed that she had been consuming nitrous oxide recreationally for over a year. The present case points out the challenges in diagnosing vitamin B12 deficiency in the context of nitrous oxide abuse due to normal concentrations of total serum vitamin B12 in a significant proportion of cases. The medical community should be aware of how difficult it can be to interpret B12 status in this specific population.

## Highlights

Vitamin B12 and holotranscobalamin may be normal in chronic nitrous oxide users

Nitrous oxide induces a defect in vitamin B12-mediated functions

Homocysteine and methylmalonic acid help identify N_2_O-users with B12 deficiency

## Introduction

The recreational use of nitrous oxide (N_2_O) as laughing gas is a growing public health concern, especially in teenagers and young adults. Nitrous oxide is inhaled for its rapid but short-lived feelings of euphoria, relaxation, and sense of detachment. Small cartridges of the gas intended to make whipped cream or larger cylinders are used to fill party balloons, from which the gas is inhaled. The popularity of N_2_O consumption is explained by its easy availability, low price, and the belief by users that it is a relatively safe and socially acceptable drug. In 2019-2020, 8.7% of 16 to 24-year-olds in United Kingdom admitted to using N_2_O (*1*). The sale of N_2_O is not subject to monitoring in most countries, only few countries or states have introduced controls (*2*).

Acute adverse effects of N_2_O use are usually mild and transient. Nitrous oxide inhalation may produce nausea, vomiting, dizziness and generalized tingling, and acute poisoning requiring medical treatment is relatively uncommon. Typically, it involves injuries from falls caused by reduced motor coordination while intoxicated. On the other hand, chronic use of N_2_O causes dose-dependent toxicity. The chronic toxicity is mainly neurological, with symptoms of peripheral neuropathy (most commonly), myelopathy, and encephalopathy (*3*, *4*). The precise mechanisms of these effects are not fully understood, but the alteration of vitamin B12 functions plays an important role (*5*). The present case report points out the challenges in diagnosing vitamin B12 deficiency in the context of N_2_O abuse due to normal concentrations of serum vitamin B12.

## Case report

A 22-year-old woman was found unconscious by a family member at home. She regained consciousness a few seconds later and was quickly brought to the emergency room of our hospital. She was found to be interactive and responsive with a Glasgow coma scale score of 13 and an oxygen saturation measured by pulse oximetry of 100%. She had no signs of visual field defect or facial weakness, and brain imaging ruled out acute stroke. Electrocardiogram and chest X-ray were free of abnormalities. Questioning revealed conduct disorder and antisocial behavior but no signs of depression.

Blood samples were collected in BD vacutainer tubes (Becton Dickinson, Franklin Lakes, USA) with lithium heparin (for routine biochemical measurements, plasma methylmalonic acid (MMA) and holotranscobalamin) and with ethylenediaminetetraacetic acid (EDTA) (for hematological tests, red blood cell folates, total homocysteine (tHcy) and genetic analyses). Complete blood count and routine biochemical parameters were analyzed using dedicated reagents on the XN-9000 (Sysmex, Kobe, Japan) and Atellica (Siemens, Erlangen, Germany) platforms, respectively. Plasma holotranscobalamin was measured by a manual ELISA (IBL International, Hamburg, Germany) using a Victor2 spectrophotometer (PerkinElmer, Wellesley, USA). Plasma concentrations of MMA and tHcy were quantified on an Acquity I-Class liquid chromatography system equipped with a Xevo TQ-S micro tandem mass spectrometer (Waters, Milford, USA) using commercial reagents (ChromSystems, Gräfelfing, Germany).

The venous blood analysis at admission was notable for macrocytic anemia (Table 1). Blood tests performed the following day confirmed the macrocytic anemia, and a peripheral blood smear found hypersegmented neutrophils. A deficiency in folates and/or vitamin B12 was therefore considered. However, serum concentrations of folate and vitamin B12, both measured by competitive immunoassays on the Atellica platform, were normal. The young woman had none of the traditional risk factors for folate deficiency (malnutrition, alcohol abuse, malabsorption *etc*.) or vitamin B12 deficiency (vegetarianism, malabsorption, proton pump inhibitors).

**Table 1:** Patient blood test results

	**Analyzer**	**Reference interval**	**Result at admission**	**Result** **the day after admission**
**Complete blood count**				
Red blood cells, x10^12^/L	Sysmex	3.80-5.80	3.23	3.26
Hemoglobin, g/L	XN-9000	115-160	98	99
Hematocrit, L/L		0.370-0.470	0.315	0.316
Mean cell volume, fL		80-100	102	102
White blood cells, x10^9^/L		4.0-10.0	4.6	4.7
Platelets, x10^9^/L		150-450	223	231
**Routine biochemical parameters**				
Sodium, mmol/L		136-145	143	144
Potassium, mmol/L		3.4-4.6	3.9	4.0
Glucose, mmol/L	Siemens	4.3-5.9	4.3	4.9
Estimated glomerular filtration rate, mL/min/1.73m^2^	Atellica	> 90	128	124
Albumin, g/L		34-50	36	N.P.
Beta-human chorionic gonadotropin, UI/L		< 6	< 2	N.P.
C-reactive protein, mg/L		< 4.0	< 4.0	N.P.
Ferritin, µg/L		8-252	N.P.	18
Serum vitamin B12, pmol/L		155-672	N.P.	253
Serum folate, nmol/L		> 9.0	N.P.	27.0
Red blood cells folate, nmol/L		> 342	N.P.	452
**Complementary tests for the evaluation of vitamin B12 status**				
Total homocysteine	Waters Xevo	5.3-20.8	N.P.	110.0
Methylmalonic acid, µmol/L	TQ-S micro	< 0.5	N.P.	7.0
Holotranscobalamin, pmol/L	Victor2	21-123	N.P.	30
N.P. - not performed.				

The presence of unexplained macrocytic anemia with normal folate and vitamin B12 concentrations prompted us to investigate further folate and vitamin B12 metabolism. As shown in Table 1, red blood cell folates and plasma holotranscobalamin (holoTC), the active form of vitamin B12, were also within the reference interval. Total homocysteine and MMA in plasma, two functional markers of vitamin B12 status, were also measured. Liquid chromatography coupled with tandem mass spectrometry revealed a tHcy concentration that was 5 times the upper limit of the reference interval (110 µmol/L), and a significantly elevated plasma concentration of MMA (7.0 µmol/L). The 4cB12 score, a combined indicator of vitamin B12 status based on age, vitamin B12, holoTC, tHcy and MMA, was calculated following the original formula developed by Fedosov and colleagues (*6*, *7*). The 4cB12 score was - 2.8 in the young woman, corresponding to a probable vitamin B12 deficiency. A large search for mutations in the hyperhomocysteinemia-related genes that encode cystathionine beta-synthase (CBS), methylmalonic aciduria and homocystinuria type C protein, and methyltetrahydrofolate reductase (MTHFR) was then ordered, and no mutation was found in the corresponding genes using a NovaSeq6000 sequencing platform (Illumina, San Diego, USA).

We investigated the possibility that this could be a false normal vitamin B12 concentration due to analytical interferences. A common interference that can lead to an overestimation of circulating concentration of vitamin B12 is due to macrovitamin B12, a complex formed by B12 with immunoglobulins (*8*). Such interference was unlikely in the present case since the serial dilution test was linear, the precipitation assay with polyethylene glycol yielding a similar result, and the vitamin B12 concentration remained normal when measured with another immunoassay (Access, Beckman Coulter, Brea, USA).

The origin of the enigmatic macrocytic anemia without decreased concentrations of vitamin B12 was clarified during the psychiatric interview, when the patient revealed that she had been consuming N_2_O recreationally for over a year. She admitted to inhaling two whipped cream charger cylinders of 640 g of pure N_2_O the day she lost consciousness, but she initially hid the information because she was worried about how her family would react. The context of N_2_O use led us to consider a functional deficiency of vitamin B12. The young woman was therefore supplemented with high-doses of vitamin B12, and blood concentrations of tHcy and MMA decreased during the follow-up (18.3 and 0.5 µmol/L, respectively, three weeks later). The author has at his disposal the patient’s informed consent, which relates to the described case report.

## Discussion

**Figure 1:**
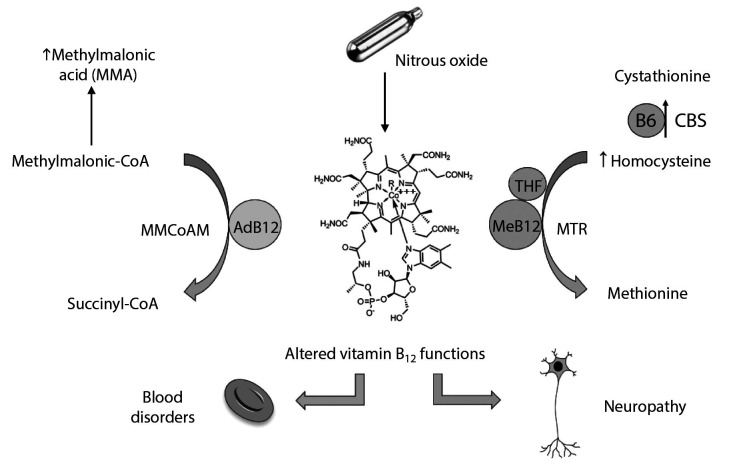
Alterations in biochemical pathways induced by nitrous oxide (N_2_O). Nitrous oxide inactivates vitamin B12 by oxidation of the cobalt atom, leading to functional defects in vitamin B12 as a cofactor for the catabolism of homocysteine and methylmalonic acid. Thus, the circulating concentrations of methylmalonic acid and homocysteine are increased in N_2_O-induced vitamin B12 deficiency. AdB12 - adenosyl-vitamin B12. CBS - cystathionine beta-synthase. MeB12 - methyl-vitamin B12. MMCoAM - methylmalonyl-CoA mutase. MTR - methionine synthase. THF - 5-methyl tetrahydrofolate.

Nitrous oxide use can impair vitamin B12 functions due to its ability to oxidize vitamin B12. In fact, vitamin B12 refers to four compounds called cobalamins, which are all characterized by a cobalt ion in the center of a corrin ring (Figure 1). Nitrous oxide inactivates vitamin B12 by oxidation of cobalt I (Co^+^) into cobalt III (Co^3+^), leading to impaired biological functions. The sixth coordination site with cobalt is variable, being either a cyano-, hydroxyl-, methyl- or 5’-deoxyadenosyl group, thus yielding cyano-, hydroxo-, methyl- (MeB12) and adenosyl- (AdB12) cobalamins, respectively. Under physiological conditions, MeB12 acts as cofactor for methionine synthase (MTR), which converts Hcy into methionine. In addition, AdB12 is a cofactor for L-methylmalonyl-CoA mutase (MMCoAM), which turns MMCoA into succinyl-CoA (Figure 1). Therefore, inactivation of vitamin B12 due to N_2_O-induced oxidation of cobalt leads to defects in MTR and MMCoAM activities, and subsequently to increased concentrations of tHcy and MMA.

The biochemical functions of vitamin B12 play an important role for DNA synthesis, hematopoiesis, and nervous system. Vitamin B12 deficiency can lead to megaloblastic anemia, fatigue, weakness and neurological signs such as numbness and tingling in the limbs. Numerous evidences suggested that the functional defect in vitamin B12 plays a major role in the pathophysiology of the neurological symptoms that occur with N_2_O use (*5*).

Vitamin B12 deficiency is usually diagnosed by measuring serum concentrations of vitamin B12, using competitive immunoassays available on most automated platforms in clinical laboratories. The present case illustrates that the recreational use of N_2_O can mask a genuine vitamin B12 deficiency if we rely only on the circulating concentrations of B12. A recent meta-analysis reported that circulating concentrations of vitamin B12 ≥ 150 pmol/L, usually considered as the threshold that defines an adequate status, were reported in 29% of cases of N_2_O abuse published in the scientific literature (*9*). In addition, Fang *et al.* recently reported normal B12 concentrations in 36% of 76 patients with N_2_O-associated peripheral neuropathy (*4*). A higher percentage of 56% in 84 subjects was reported in a recent systematic review (*10*).

Other biomarkers must be considered to accurately assess vitamin B12 status in N_2_O users. Firstly, holoTC, also called bioactive vitamin B12, should be of interest. Approximately one-quarter of circulating vitamin B12 binds to the specific transport protein transcobalamin to yield holoTC, and the remaining vitamin B12 is carried by haptocorrin to form holohaptocorrin. HoloTC is able to transport vitamin B12 into the cells by binding to a specific receptor, unlike holohaptocorrin which is biologically inactive. Circulating concentrations of holoTC can be measured by immunoassays, and are sometimes considered more sensitive than total vitamin B12 for screening vitamin B12 deficiency (*7*). Data are scarce in the literature, but normal concentrations of holoTC were also reported in all of the four tested N_2_O users, who exhibited neurological symptoms despite normal total vitamin B12 concentrations (*11*). In addition, an Australian study reported than 11 of the 17 N_2_O users with myeloneuropathy also had normal concentrations of holoTC, while half had decreased total vitamin B12 (*12*).

Total homocysteine is another biomarker of vitamin B12 status which must be considered in the context of N_2_O use. In fact, vitamin B12 deficiency alters MTR activity, leading to increased circulating concentrations of tHcy (Figure 1). Homocysteine is a sulfur-containing amino acid derived from the catabolism of the essential amino acid methionine into cysteine. Hyperhomocysteinemia is usually classified as mild (15-30 μmol/L), intermediate (30-100 μmol/L) or severe (> 100 μmol/L) according to the circulating concentrations of tHcy (*13*). The severe form of hyperhomocysteinemia observed in the present case is rare and is typically due to inherited defects in Hcy metabolism or profound deficiencies in vitamins B6, B12 and/or folate, which are cofactors of enzymes catabolizing Hcy (*13*). Specifically, vitamin B6 is a cofactor of CBS, while vitamin B12 and folate are required for MTR activity. Severe hyperhomocysteinemia can be also due to gene defects in enzymes involved in the metabolism of methionine and Hcy (*14*). The three most frequent genetic causes of severe hyperhomocysteinemia are CBS deficiency (classical homocystinuria), methylmalonic aciduria with homocystinuria cblC type, and severe deficiency in MTHFR, which is the enzyme that generates the active form of folate (5-methyltetrahydrofolate) for MTR activity (*14*). In the present case, although the results were obtained after the puzzle was solved, no mutation was found in the corresponding genes. It has been reported that 93% of N_2_O users with neurological disorders had elevated concentrations of tHcy (*15*). Similarly, 87% of N_2_O users with neurological signs had increased plasma concentrations of tHcy, while only 35% of them had low concentrations of vitamin B12 (*16*).

Methylmalonic acid is also a functional marker of vitamin B12 deficiency. In fact, vitamin B12 deficiency impairs MMCoAM activity, which results in increased conversion of MMCoA into MMA (Figure 1). However, cautions must be taken since MMA concentrations are not only elevated in vitamin B12 deficiency. They are also known to be increased during renal dysfunction (like for tHcy) or inborn errors of metabolism due to mutation in MMCoAM or by deficient synthesis of AdB12 (methylmalonyl acidemia). In the case we reported here, the elevation in both tHcy and MMA concentrations without renal impairment strongly guided the diagnosis towards vitamin B12 functional deficiency. It has been shown that 100% of N_2_O users had increased concentrations of MMA in two cohorts of N_2_O users with neurological disorders (*17*, *18*). The combination of elevated concentrations of both tHcy and MMA is sensitive since it had been found that 90% of N_2_O users had both tHcy > 15 µmol/L and MMA > 0.4 µmol/L (*9*).

Lastly, it has been proposed a combined indicator of vitamin B12 status, called 4cB12, in order to improve the detection of subclinical B12 deficiency in the general population (*6*, *7*). The calculation of 4cB12 is based on circulating concentrations of total vitamin B12, holoTC, tHcy and MMA (*6*). Assessing vitamin B12 status with 4cB12 therefore leads to significant additional costs and time. Although the 4cB12 score was validated in a context other than N_2_O abuse, it may be also more sensitive than vitamin B12 alone in N_2_O abuse (*6*, *7*). Indeed, 91% of N_2_O users have altered B12 status based on the 4cB12 score whereas only 71% exhibit vitamin B12 concentrations < 150 pmol/L (*9*). In the young woman discussed here, the 4cB12 score indicated a probable vitamin B12 deficiency.

To conclude, the present case points out the challenges in diagnosing vitamin B12 deficiency in the context of N_2_O abuse due to normal concentrations of total serum vitamin B12 in around one-third of cases. The medical community should be aware of how difficult it can be to interpret B12 status in this specific population, in particular due to the increasing trend of recreational N_2_O use in Western countries. Obviously, diagnosing vitamin B12 deficiency is even more challenging in individuals who are reluctant to admit to N_2_O consumption for fear of family judgment. Here, collecting accurate information during medical interview would have avoided unnecessary efforts and costs (> 500 euros in the present case) related to additional tests. A proper evaluation of a patient with a suspicion of N_2_O-induced toxicity should include a rigorous laboratory evaluation, which consists of a complete blood count, folate, total vitamin B12, tHcy, and MMA.

## Data Availability

The data generated and analyzed in the presented study are available from the corresponding author on request.
